# Proteinuria in Alport Syndrome: Treatment With Sodium-Glucose Co-Transporter-2 (SGLT2) Inhibitors

**DOI:** 10.7759/cureus.83455

**Published:** 2025-05-04

**Authors:** Gautam Agrawal, Bhawna Agarwal, Pallavi Shirsat, Kunal Sonavane

**Affiliations:** 1 Nephrology, Independence Health System, Greensburg, USA; 2 Internal Medicine, University of Pittsburgh Medical Center McKeesport Hospital, McKeesport, USA; 3 Nephrology, Minden Medical Center, Minden, Louisiana, USA; 4 Internal Medicine, Willis Knighton Medical Center, Bossier City, USA

**Keywords:** alport syndrome, asymptomatic hematuria, autosomal dominant inheritance, sodium-glucose cotransporter-2 (sglt2) inhibitors, subnephrotic proteinuria

## Abstract

Alport syndrome (AS) is a genetic disorder characterized by progressive kidney disease, hearing loss, and eye abnormalities. It is caused by mutations in the genes responsible for producing type IV collagen, which is a crucial component of the glomerular basement membrane, the cochlea, and the lens of the eye. We present a case of a 40-year-old female who presented with persistent microscopic hematuria and proteinuria and was diagnosed with autosomal dominant AS based on kidney biopsy and genetic testing. This case report discusses the clinical presentation, diagnostic work-up, and management approach of patients with AS. We highlight the current advancement in management of CKD with SGLT 2 inhibitors, with limited research regarding the benefit of sodium-glucose cotransporter-2 inhibitors (SGLT2i) in patients with genetic disorders like AS. Early recognition and management of AS with ACE inhibitors and SGLT2i are vital to prevent irreversible kidney damage and other complications. Genetic testing and multidisciplinary care play key roles in the treatment of AS.

## Introduction

Alport syndrome (AS), also known as hereditary nephritis, is a hereditary disorder characterized by progressive renal disease, sensorineural hearing loss, and ocular abnormalities. It is caused by mutations in the COL4A3-5 genes, which encode the alpha3-5 chains of type IV collagen, a crucial component of the glomerular basement membrane (GBM) in kidneys, the cochlea in the ear and the retina in the eye. The mode of inheritance can be X-linked, autosomal dominant, autosomal recessive, or rarely digenic inheritance. AS is the most common cause of inherited kidney disease and the second most common genetic cause of kidney failure [1,2]. Renal manifestations include hematuria, proteinuria, hypertension, and progression to kidney failure. Diagnosis is confirmed by molecular genetic testing or skin or kidney biopsy. 

Despite the increasing availability of genetic testing, tissue studies remain essential for evaluating Alport syndrome (AS) in patients with persistent hematuria. Kidney biopsy findings often reveal thinning of the glomerular basement membrane [3]. Currently, there is no specific cure for AS, and patients who develop renal failure typically require kidney transplantation. The standard of care to slow the progression of renal deterioration involves treatment with angiotensin-converting-enzyme (ACE) inhibitors [3]. Initiating therapy at a higher glomerular filtration rate (GFR) has been shown to delay the onset of kidney failure. Sodium-glucose cotransporter 2 (SGLT2) inhibitors can reduce albuminuria and slow the progression of chronic kidney disease [4], although data regarding their efficacy in patients with AS remains limited.

This case report discusses the clinical presentation, diagnostic workup, and management of AS in a 40-year-old female patient. It highlights ongoing research in interventions to slow renal disease progression and the need for further research regarding the use of sodium-glucose cotransporter-2 inhibitors (SGLT2i) in patients with AS.

## Case presentation

This is the case of a 40-year-old female who had pertinent past medical history of a solitary episode of kidney stone (which she passed spontaneously), irritable bowel syndrome, depression, and a ruptured ovarian cyst. She denied any known family history of hereditary kidney disease. 

She was found to have persistent proteinuria and microscopic hematuria, for which she was seen in the nephrology clinic. On physical examination, her weight was 210 lbs, and her blood pressure was 120/78 mm Hg. She appeared euvolemic on examination, with no peripheral edema. Her lungs were clear to auscultation bilaterally, and her heart sounds were regular. She had no rash, lymphadenopathy, oral ulcers, or tattoos. As shown in Table 1, her laboratory values demonstrated sub-nephrotic proteinuria, with a 24-hour protein level of 2,141 milligrams. She had normal renal function, with a serum creatinine of 0.7 mg/dL. Serological workup showed a negative anti-nuclear antibody, negative anti-double-stranded DNA antibody, normal complement levels, and a serum protein electrophoresis that was negative for a monoclonal spike. Her hemoglobin A1C was 5.1%. 

**Table 1:** Laboratory Values ANA: Antinuclear antibody; Anti-ds: Anti-double stranded.

Laboratory tests	Results	Reference range
24-hour protein	2141	< 150 mgs
Serum albumin	3.8	3.5-5.7 gm/dl
Blood urea nitrogen (BUN)	9	7-25 mg/dl
Creatinine	0.7	0.6-1.2 mg/dl
Calcium	9	8.6 - 10.3 mg/dl
Hemoglobin	13	11.7-15.8 gm/dl
ANA	0.42	<0.8-Negative
Anti-ds DNA	Negative	<25-Negative
Hemoglobin A1c	5.1	5.7-6.4 (Prediabetic) %
Hepatitis B surface antigen	Non-reactive	Non-reactive
Hepatitis C antibody	Non-reactive	Non-reactive

**Figure 1:**
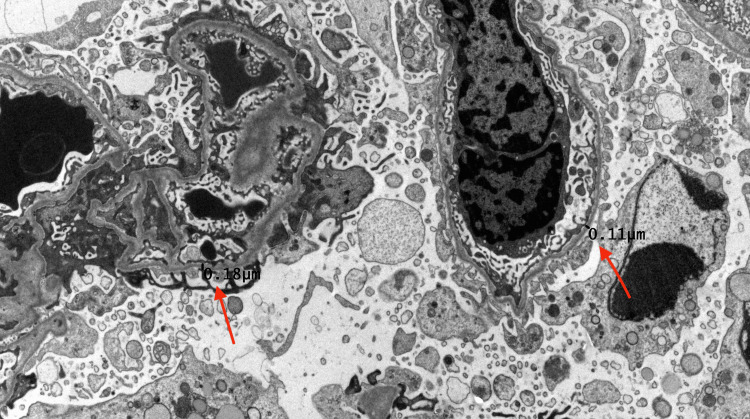
Kidney biopsy slide showing thinning of glomerular basement membrane Red arrow indicating glomerular basement membrane measurements.

She was referred for a kidney biopsy, which revealed diffuse thinning of the glomerular basement membrane as shown in Figure 1, with a mean thickness of 178 nanometers (normal range of 250-330 nanometers), raising the possibility of Alport syndrome. 

Genetic testing showed a monoallelic mutation in the COL4A4 gene, which was likely pathogenic and consistent with an autosomal dominant inheritance pattern. She was started on lisinopril, which was gradually up-titrated to the maximum tolerated dose. Her proteinuria improved to 1246 mg in the 24-hour urine test. She was then started on dapagliflozin for renal protection. She has tolerated dapagliflozin well, with no episodes of urinary tract infections, hypotension, or dehydration. Her proteinuria has fluctuated but improved overall, with the 24-hour protein level of 1007 mg after one year of therapy. Her renal function has remained stable, with a serum creatinine of 0.7 mg/dL nearly three years post-treatment.

## Discussion

This case underscores the importance of considering AS in patients with unexplained hematuria or proteinuria, particularly when there is a family history of similar symptoms. Early diagnosis and comprehensive management are essential to slow the progression of kidney disease and prevent complications. AS is a genetically and phenotypically heterogeneous disorder. Clinical presentations can be variable depending on the mode of inheritance or genetic variant. X-linked Alport syndrome commonly results in end-stage renal disease (ESRD) in males, affecting approximately 50% by age 25 and 90% by age 40 [2]. Over 90% of patients with autosomal recessive AS progress to end-stage kidney disease (ESKD) by the age of 40 [2]. Patients with autosomal dominant usually have a slower progression to ESRD. 

Early intervention with ACE inhibitors or angiotensin receptor blockers (ARBs) can reduce proteinuria and delay the progression of renal disease [3], as noticed in our patient. SGLT2i reduces albuminuria independently of glucose control by lowering tubular glucose reabsorption, blood pressure, and intraglomerular pressure, key factors in slowing chronic kidney disease (CKD) progression [4]. However, the efficacy of SGLT2i in underrepresented subgroups, as patients with AS or immune-mediated glomerular disorders, remains uncertain, as they may affect lean individuals whose CKD progression may involve alternative pathways like inflammation or genetic abnormalities [5]. Some evidence suggests that the renal benefits of SGLT2i may be influenced by body mass index (BMI), with greater effects observed in patients with higher BMI [5]. Given these considerations, dedicated clinical trials are necessary to assess the efficacy of SGLT2i in patients with this patient population. 

A case series involving patients with hereditary podocytopathies, including Alport syndrome, reported that treatment with SGLT2i significantly reduced the urinary albumin-creatinine ratio (UACR), indicating a potential benefit in reducing proteinuria and slowing renal dysfunction [6]. However, due to the limited treatment duration and small sample size, more robust studies are needed to validate these results. There is an ongoing trial named the DOUBLE PRO-TECT Alport Trial, which is a multicenter, randomized, double-blind, placebo-controlled trial that aims to assess the safety and efficacy of dapagliflozin, an SGLT2i, in young patients with Alport syndrome. The primary outcome is the change in UACR from baseline to week 48, with secondary outcomes including changes in estimated glomerular filtration rate (eGFR) [7]. Renal transplantation is the definitive treatment for ESRD. Given the genetic nature of the disease, counseling for the patient and their family is essential.

## Conclusions

Early intervention with ACE inhibitors or angiotensin receptor blockers (ARBs) is recommended to reduce proteinuria and slow the progression of renal disease in Alport syndrome. However, the efficacy of SGLT2 inhibitors (SGLT2i) in underrepresented subgroups, such as patients with Alport syndrome, remains uncertain. This case highlights the importance of early recognition and diagnosis of Alport syndrome to enable timely therapeutic intervention with dual therapy using ACE inhibitors and SGLT2i, aiming to slow disease progression and prevent complications. Further research and long-term data are needed to better understand the use of SGLT2i in patients with Alport syndrome.

## References

[1] Savige J (2025). Alport syndrome: an update. Curr Opin Nephrol Hypertens.

[2] Chavez E, Goncalves S, Rheault MN, Fornoni A (2024). Alport syndrome. Adv Kidney Dis Health.

[3] Kashtan CE (2021). Alport syndrome: achieving early diagnosis and treatment. Am J Kidney Dis.

[4] (2024). KDIGO 2024 Clinical Practice Guideline for the evaluation and management of chronic kidney disease. Kidney Int.

[5] Romagnani P (2025). SGLT2 inhibitors in CKD: are they really effective in all patients?. Nephrol Dial Transplant.

[6] Boeckhaus J, Gross O (2021). Sodium-glucose cotransporter-2 inhibitors in patients with hereditary podocytopathies, alport syndrome, and FSGS: a case series to better plan a large-scale study. Cells.

[7] Gross O, Boeckhaus J, Weber LT (2025). Protocol and rationale for a randomized controlled SGLT2 inhibitor trial in paediatric and young adult populations with chronic kidney disease: DOUBLE PRO-TECT Alport. Nephrol Dial Transplant.

